# Ensuring Indigenous co-leadership in health research: a Can-SOLVE CKD case example

**DOI:** 10.1186/s12939-023-02044-9

**Published:** 2023-11-08

**Authors:** Cathy Woods, Craig Settee, Mary Beaucage, Helen Robinson-Settee, Arlene Desjarlais, Evan Adams, Catherine Turner, Malcolm King, Letitia Pokiak, Mary Wilson, Evelyn Voyageur, Chantel Large, Jonathan McGavock, Joanne Kappel, Helen Chiu, Tamara Beardy, Isabelle Flett, James Scholey, Heather Harris, Jocelyn Jones, Latash Maurice Nahanee, Delhia Nahanee, on behalf of the Can-SOLVE CKD Indigenous Peoples’ Engagement, Research Council (IPERC), Mary Beaucage, Arlene Desjarlais, Cathy Woods, George Fontaine, Malcolm King, Evelyn Voyageur, Jonathan McGavock, Tamara Beardy, Donna Saucier, Isabelle Flett, Darrell Ross, Tannyce Cook, Letitia Pokiak, Latash Maurice Nahanee, Delhia Nahanee, Joanne Kappel, Allison Dart

**Affiliations:** 1 Canadians Seeking Solutions and Innovations to Overcome Chronic Kidney Disease (Can-SOLVE CKD), Vancouver, Canada; 2 Canadian Donation and Transplantation Research Program, Edmonton, Canada; 3 First Nations Health Authority (BC), Vancouver, Canada; 4 Community Health and Epidemiology, University of Saskatchewan, Saskatoon, Canada; 5 Saskatchewan Centre for Patient-Oriented Research, Saskatoon, Canada; 6 Department of Pediatrics and Child Health, DREAM Research Theme, Rady Faculty of Health Sciences, University of Manitoba, Winnipeg, Canada; 7 University of Saskatchewan, Saskatoon, Canada; 8 BC Renal, Vancouver, Canada; 9 Diabetes Action Canada, Toronto, Canada

**Keywords:** Indigenous health, Self-determination, Research, Health equity, Kidney disease

## Abstract

**Background:**

Indigenous people are insightful and informed about their own health and wellness, yet their visions, strengths and knowledge are rarely incorporated into health research. This can lead to subpar engagement or irrelevant research practices, which exacerbates the existing health inequities Indigenous people experience compared to the non-Indigenous population. Data consistently underscores the importance of Indigenous self-determination in research as a means to address health inequities. However, there are few formal methods to support this goal within the existing research context, which is dominated by Western perspectives.

**Main text:**

Canadians Seeking Solutions and Innovations to Overcome Chronic Kidney Disease (Can-SOLVE CKD) is a patient-oriented research network in Canada that recognizes the need to create the space to facilitate Indigenous self-determination in research. Indigenous members of the network therefore created and evolved a unique group, called the Indigenous Peoples' Engagement and Research Council (IPERC). IPERC plays a critical role in informing Can-SOLVE CKD research priorities, as well as creating tools to support Indigenous-specific research and engagement. This approach ensures that Indigenous voices and knowledge are critical threads within the fabric of the network’s operations and research projects. Here, we describe the methods taken to create a council such as IPERC, and provide examples of initiatives by the council that aim to increase Indigenous representation, participation and partnership in research. We share lessons learned on what factors contribute to the success of IPERC, which could be valuable for other organizations interested in creating Indigenous-led research councils.

**Conclusion:**

Indigenous self-determination in research is critical for addressing health inequities. Here, we present a unique model, led by a council of diverse Indigenous people, which could help reduce health equities and lead to a better era of research for everyone.

## Background

The health needs of one population can be very different from the needs of another, especially when historical and cultural context is considered. Indigenous peoples in Canada, and other colonized countries, experience health inequities related to colonialism and on-going structural racism that are unique, complex and challenging to address. Centuries of racist policies, oppression, forced assimilation and intergenerational trauma have had a direct and detrimental effect on the health and well-being of Indigenous peoples, resulting in higher incidence and prevalence rates of many chronic diseases compared to the general population [1–4]. These health inequities are compounded by a pervasive bias in the way settler scientists design and conduct research. Settler-driven research is often grounded in individualistic paradigms and “Western” views about health. As a result, research regarding Indigenous health is often biased towards deficit-based frameworks [5, 6], ignores the impacts of historical and on-going structural oppression, lacks cultural appropriateness, and does not adequately address the needs of Indigenous peoples.

Many studies have identified ways of addressing inequities in health among Indigenous people, which in large part, involve ensuring Indigenous self-determination, participation and leadership in health research [4, 7, 8]. In particular, it is important to ensure that research is grounded in frameworks that account for the unique cultural contexts and histories Indigenous peoples – which include acknowledging the impact of structural oppression and colonization while incorporating Indigenous teachings, traditions and holistic approaches to well-being.

Indigenous self-determination and leadership in research has numerous benefits, and should be considered an inherent right [9]. Historically, research is designed, funded and conducted unilaterally by non-Indigenous researchers, whereby little to no consultation or shared power with Indigenous communities takes place throughout the various stages of a research project. Such unilateral approaches do not account for Indigenous ways of knowing, being and doing. This is especially problematic for research that aims to improve health outcomes of Indigenous people, who practice a more holistic approach to health and wellness that extends beyond a physical state, to include spiritual, emotional, mental and mindful well-being. Research taken through solely a Western approach can easily overlook the full scope of Indigenous health and wellness.

As well, numerous unilateral studies conducted by non-Indigenous people in the past have been profoundly unethical and harmful to Indigenous peoples [10]. Given these historical offenses, as well as modern forms of individual, structural and institutional racism that Indigenous people currently face in the health care system, it is unsurprising that Indigenous people are disproportionately more likely to mistrust medical science and the health care system compared to ethnic majority populations [11, 12].

Indigenous leadership in research is a basic step that can be taken to foster more trust in research among Indigenous people, and ensure that studies are ethical and align with their interests. Both strong community participation and methods that acknowledge Indigenous ways of knowing have been identified as an important means for addressing health disparities [13]. While the benefits of Indigenous leadership in research are clear, it’s important to note that current health care and research institutions are inherently Westernized, which makes it difficult to create the space Indigenous people need to be leaders in research. While the research landscape is evolving to facilitate better inclusion and engagement of Indigenous people in the research process, this trend is still relatively new and it could be beneficial for health researchers to learn about case studies of meaningful and respectful engagement.

**Fig. 1:**
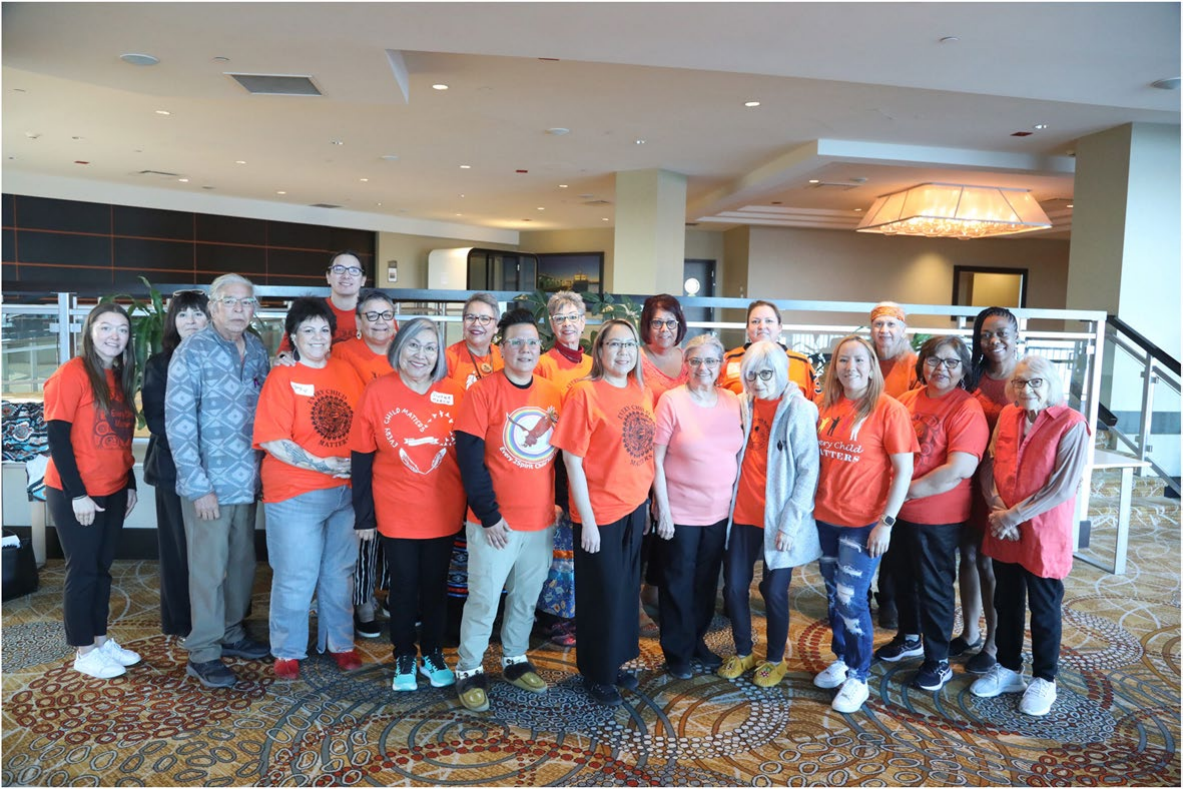
IPERC at Can-SOLVE CKD’s annual meeting in 2023

Canadians Seeking Solutions and Innovations to Overcome Chronic Kidney Disease (Can-SOLVE CKD) [14] is a patient-oriented research network in Canada that sought to create a space for Indigenous leadership within its network. With commitment and support of the network leadership and a desire to address health inequity in Indigenous communities, Indigenous members of the network therefore created and evolved a unique group, called the Indigenous Peoples' Engagement and Research Council (IPERC). IPERC consists of Indigenous patient partners, scholars, and policy-makers, who play a critical role in informing Can-SOLVE CKD research priorities, as well as creating tools to support Indigenous-specific research and engagement (Fig. 1).

Through the unique structure of IPERC, Indigenous and non-Indigenous members of the Can-SOLVE CKD network work together to adopt *Etuaptmumk* (the Mi’kmaq word for a two-eyed see approach) [15], where both Western and Indigenous perspectives and knowledge are equally respected and valued. This approach ensures that Indigenous voices and knowledge are woven into the network’s operations and research projects. Special emphasis is given to IPERC member voices in guiding the development and implementation of Indigenous-specific strategies and projects across the network. Importantly, members of IPERC are engaged in all stages of the research process, from the design of studies through to the implementation phase, mobilizing Indigenous agency and self-determination.

Here, we describe the methods taken to create a council such as IPERC, and provide examples of initiatives by the council that aim to increase Indigenous representation, participation and partnership in research. We share lessons learned on the successes and challenges experienced by IPERC, which could be valuable for other organizations interested in creating Indigenous-led research councils to facilitate Indigenous self-determination in research. An ethnographic narrative description of creating an Indigenous patient-led council is provided.

### IPERC: engagement process

**Fig. 2:**
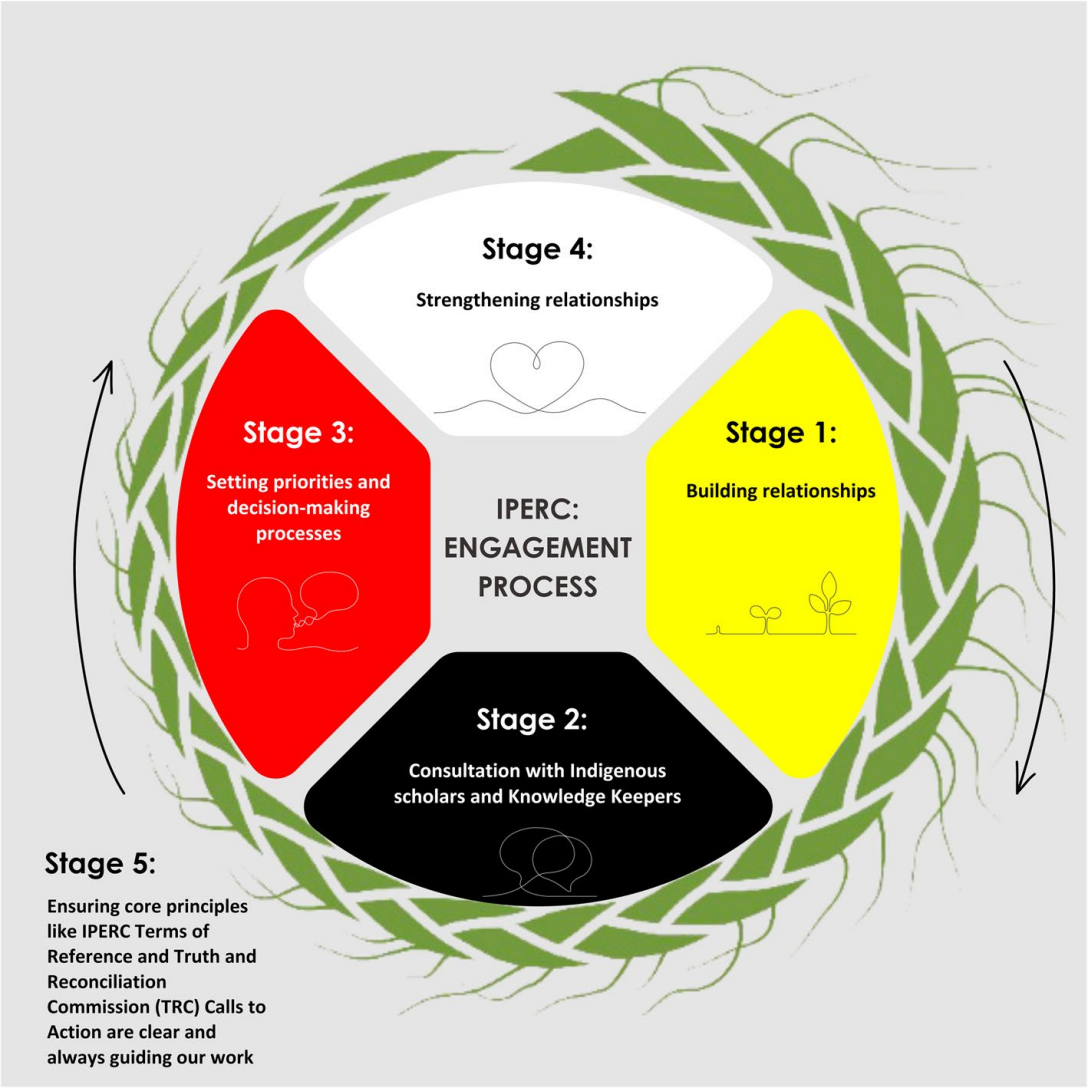
The five stages of forming and sustaining IPERC include: building relationships, consulting with Indigenous scholars and Knowledge Keepers, setting priorities and decision-making processes, strengthening relationships and using guiding principles, such as the IPERC terms of reference and Truth and Reconciliation Calls to Action

The creation of the IPERC council was an operative, relationship-based process. Funding for Can-SOLVE CKD was secured in 2015 through the Canadian Institutes of Health Research’s (CIHR’s) Strategy for Patient Oriented Research initiative. As a network funded specifically to advance patient-oriented research, it was understood from the beginning that patients would play a vital role at the centre of the organization, co-leading at all stages of research and other projects. Figure 2 describes the process of creating IPERC, and the guiding principles behind its ongoing work.

#### Stage 1- Building relationships

A small handful of Indigenous patient partners, health professionals and scholars were involved during the priority-setting workshop before the network was formed. These individuals identified health inequities as a priority, and emphasized the need for Indigenous-specific research projects, as well as resources dedicated to supporting a culturally safe environment. The idea for an Indigenous-specific council was identified during the priority-setting workshop and became part of the grant application and work plan of the network, and it eventually came to fruition.

#### Stage 2 – Consultation with Indigenous scholars and knowledge keepers

To establish the council, early founding members sought the expertise of Indigenous scholars and Knowledge Keepers who bring a wealth of Indigenous knowledge from various traditions and could provide guidance on how to establish and grow a unique Indigenous-led research council. Some recommendations from these experts that were incorporated into practice include allocating dedicated funds and staff to facilitate appropriate Indigenous-specific initiatives by the network, as well as cultural competency training for all staff involved with any Indigenous engagement.

#### Stage 3 – Setting priorities and decision-making processes

Early on, IPERC members decided that the council would make decisions on a consensus basis. As well, all decisions on Indigenous-specific initiatives were to be based on feedback from IPERC – in line with the concept of “nothing about us, without us” [16]. 

#### Stage 4 – Strengthening relationships

To foster more awareness of IPERC and recruit additional members, founding members attended conferences and gave presentations. Especially through connections of founding members and by attending Indigenous-specific events, more members were recruited over time. Whereas IPERC had about 10 members when it first formed, it has since grown to include 22 members. Importantly, IPERC members are from various First Nations, Inuit and Métis communities, reflecting a diverse array of Indigenous voices and perspectives.

#### Stage 5 – Creating a terms of reference

In IPERC’s established Terms of Reference, the document points to three key Truth and Reconciliation Commission (TRC) calls to action: #18, #19, #22 and #23 [17]. These include understanding and acknowledging that the health of Indigenous people is a direct result of previous Canadian government policies; working to close the gaps in health outcomes between Indigenous and non-Indigenous communities within chronic diseases; ensuring that the health care system recognizes the value of Indigenous healing practices and incorporates these into care when appropriate; and to increase the number Indigenous professionals working and involved in the health care field and providing cultural competency trainings.

### Outcomes of IPERC

#### Pathway for ethical engagement

Shortly after Can-SOLVE CKD was created, an Indigenous member of IPERC, Helen Robinson-Settee, saw a need for specific educational resources to support non-Indigenous research team members in developing cultural competency and creating culturally safe spaces in health research. Following a visioning workshop, Robinson-Settee partnered with Indigenous leaders at other health research institutions to create the *Wabishki Bizhiko Skaanj* (pronounced wah-bish-kih biish-ih-goo skaa-nch and meaning “White Horse” in Anishinaabemowin) learning pathway, an Indigenous cultural competency training program dedicated to distilling racism in health research and care [18]. All Can-SOLVE CKD members are encouraged to partake in the pathway, and the success of the program has gained the attention of other health institutions and organizations.

#### Informing research related to kidney health

In its first five years, IPERC has grown and evolved to be an important influence within the Can-SOLVE CKD network and beyond. The network funded and coordinated 18 research projects in its first phase, and nine research projects in its second phase, and members of IPERC act as a patient partner on nearly every project.

Three Can-SOLVE CKD research projects are focused specifically on Indigenous health. Guidance from IPERC members has helped ensure that the network and project leaders consider how Indigenous peoples and communities may be impacted by the projects, and how these projects align with Indigenous values and priorities.

#### Changing network culture

Along with guiding studies and other projects within Can-SOLVE CKD, IPERC has helped create a beneficial and significant culture shift within the network. A small circle and several individual interviews took place to reflect on the role of IPERC and how it has progressed over the years. One member notes that a culture shift in a large, predominantly non-Indigenous organization is not easy, but that the council offers a “wonderful model and process for Indigenous people in health care.” Members in the small circle agreed that IPERC has helped Indigenous voices be heard throughout the network.

#### Empowerment and decision making within the network

Over time, more Indigenous traditions were woven into IPERC operations, as well as Can-SOLVE CKD’s broader operations and research projects. For example, land acknowledgments are now shared at the beginning of all major meetings as a reminder of Indigenous ancestral stewardship of the lands and traditional territories we gather on, and to acknowledge the impact of colonialism, among other important reasons. As well, a number of Indigenous ceremonies have taken place at network events, including sharing circles, smudging, cedar brushings, tobacco offerings, sweat lodges, naming ceremonies and blanketing ceremonies. Elders/Knowledge Keepers – key leaders in Indigenous ways of knowing, being and doing – are often invited to share their knowledges or blessings during meetings. These practices may help to create culturally safe spaces for IPERC members to thrive within. As well, IPERC alternates between business meetings, and Tea and Bannock meetings, the latter of which offers members a chance to connect socially and cultivate closer relationships.

Importantly, leadership and guidance from IPERC, as well as use of the *Wabishki Bizhiko Skaanj* learning pathway, has helped create a shift in approaches, attitudes and understanding among many non-Indigenous members of the network, who are now more likely to practice *Etuaptmumk* (two-eyed seeing), ensuring that research is rooted in mutual respect [16].

#### Knowledge translation

The successful model that IPERC embodies has been gaining the attention of other research organizations. Members of IPERC have presented their approach to research at numerous conferences nationally and internationally, including those hosted by the Canadian Society of Nephrology, American Society of Nephrology, World Congress of Nephrology, and the World Indigenous Peoples' Conference on Education.

### The experiences of Indigenous patient partners and researchers

*“We realized we needed to build relationships and trust in order to succeed, so we did this through ceremony, education and partnership.” – Cathy Woods, founding IPERC co-chair*

#### Testimonial #1 – Indigenous IPERC member

We realized we needed to build relationships and trust in order to succeed, so we did this through ceremony, education and partnership. The learning pathway was created to provide a safe space for all.

*I’m so amazed when you look at the relationships we fostered with the leaders and researchers and all involved in the network. For me to be able to see this small group of Indigenous folks with this massive mandate accomplishing all these tasks within a few years is overwhelming. People come to us for advice, letters of endorsements and truly want to understand how we did this so well. It was a lot of hard work and we were able to build relationships within the kidney research community. Through this process I was able to find my voice and give back to my community who supported me to deal with my kidney disease.*

*Cathy Woods, Naicatchewenin First Nation, founding co-chair IPERC*

#### Testimonial #2 – Indigenous IPERC member

*“IPERC really gave me confidence that a partnership between the health system and Indigenous people can work – that they can be within the system and have their voice heard.” – Dr. Evan Adams, founding IPERC co-chair*

*I became involved with IPERC because it was important as a distinction-based approach to healthcare and research for Indigenous peoples. I am First Nations and have very distinct experiences in this country and its healthcare system. Lots of Indigenous people think the healthcare system is not meant for them. I just saw room for improvement for research and health to be inclusive of Indigenous patients but also Indigenous views, so that [we] can come into the system and feel confident about it… IPERC really gave me confidence that a partnership between the health system and Indigenous people can work – that they can be within the system and have their voice heard.”*

*Dr. Evan Adams, Tla’amin First Nation, founding IPERC co-chair*

#### Testimonial #3 – Indigenous IPERC member

*“IPERC has helped shape the culture of Can-SOLVE CKD and our way of doing things has been sought out by other research groups across Canada as a solid model for Indigenous partnerships in research.” – Mary Beaucage, IPERC member*

*When we started out in 2016, I really had no idea what our goal was. As we started getting to know each other with every meeting, we started to gel and create a vision. It’s amazing how far we’ve come in 7 years. IPERC has helped shape the culture of Can-SOLVE CKD and our way of doing things has been sought out by other research groups across Canada as a solid model for Indigenous partnerships in research. Those are the legacies I’m proud of.*

*Mary Beaucage, Nipissing First Nation, IPERC member and former co-chair*

#### Testimonial #4 – Non-Indigenous Can-SOLVE CKD member

Engagement with IPERC has enabled me to gain a greater understanding and awareness of the tragic history that has defined the relationship between settlers and Indigenous people in Canada. Moreover, I have benefitted and continue to benefit from the insights, wisdom, and scholarship generously provided by our IPERC members and by our Indigenous co-leads in the research network as we seek to lessen the burden of chronic kidney disease.

*I have always considered myself “a student of history” and yet I was woefully unaware of much that has happened in Canada – history that I am now painfully aware of as we work together. This partnership with IPERC has changed me and it has energized me as we work towards addressing inequities in kidney health care in Canada.*

*Jim Scholey, Can-SOLVE CKD Leadership Team*

## Discussion

Creating an Indigenous-led council has helped ensure that Indigenous voices are critical threads of fabric within Can-SOLVE CKD. Indeed, multiple IPERC members have noted that the council has helped them find a voice in the research landscape, and agree it has led to improved self-determination and leadership in Indigenous health and research. It facilitates the concept of “research for us, by us.” Using the analogy of thread and fabric, one member notes that a new pattern is being stitched – whereas research can be considered a fabric that has traditionally been “stitched” with a Westernized pattern, IPERC is helping to create a completely new pattern that embodies both Western and Indigenous threads.

The inclusion of voices and the way in which the network conducts itself has changed dramatically over the years. The incorporation of land acknowledgments and ceremony is very noteworthy. For example, one IPERC member attended a meeting with a different research organization that did not open with a land acknowledgment, which accentuated her sense of power imbalances between Indigenous and non-Indigenous people. Land acknowledgments are therefore of upmost importance for helping to establish a culturally safe space for Indigenous people at Can-SOLVE CKD meetings—they are acts of reconciliation. IPERC created a Land Acknowledgment Teaching series, [19] available freely online to anyone, to support people in understanding the importance of land acknowledgments.

While IPERC offers numerous benefits, there are several challenges that come with this model, however. As one IPERC member notes, Indigenous people are “stretched thin” in their commitments. This member contributes as a patient partner with a second research institution as well. It is important to note that, whether patient partners are volunteers or compensated, acting as a patient partner can require significant amounts of time and energy. Special care should be taken to avoid burning out patient partners.

It is especially important to keep in mind the historical context and legacy of colonization, and the risks of re-traumatizing or exacerbating emotional labour of Indigenous individuals. Adequate support is essential—this includes Knowledge Keeper support, ceremony and adopting a trauma-informed approach to engagement.

As well, any approach involving collaboration and input from many people can take more time than expected. It is therefore helpful to be prepared to deviate from tightly timed agendas, in order to ensure that a topic is given sufficient time to be discussed as needed.

Lastly, Can-SOLVE CKD is, at its heart, a patient-oriented research network. It is therefore able to dedicate much time and resources to ensuring input from patient partners. The ability to delve fully into patient-oriented research – where patient partners are the key drivers of all research priorities – may be difficult for other research institutions to implement without adequate funding and other resources. As IPERC and its role grew within the network, we were able to hire an IPERC coordinator to oversee the council’s needs, coordinate meetings, and arranged ceremonies and teachings with Elders and Knowledge Keepers. An Indigenous Initiatives team, consisting of four Indigenous people, also helps oversee relevant operations across the network.

## Conclusion

Indigenous people have endured extreme challenges related to colonialism that have profoundly negatively affected their health and well-being, resulting in health inequities. These inequities are compounded by the fact that research often excludes Indigenous people or is conducted without appropriate cultural context or consultation. As more and more research entities reach out to Indigenous people for input on matters related to Indigenous health, it is important to consider culturally safe, respectful and meaningful ways of engagement. Within our research network, we’ve found that engaging a council of Indigenous patient partners researchers, health professionals, and policy-makers has been highly beneficial in advancing Indigenous leadership and self-determination in research. Importantly, this council has also helped create a shift in approaches, attitudes and understanding among many non-Indigenous members of the network, fostering more culturally competent members and creating more culturally safe spaces for a *Etuaptmumk* (a two-eyed seeing) approach to research that is rooted in mutual respect. We believe more research entities should consider creating similar Indigenous-led councils within their networks, which could help reduce health equities and lead to a better era of research for everyone.

## Data Availability

Data sharing is not applicable to this article as no datasets were generated or analysed during the current study.

## Abbreviations

CIHR: Canadian Institutes of Health Research
Can-SOLVE CKD: Canadians Seeking Solutions and Innovations to Overcome Chronic Kidney Disease
IPERC: Indigenous Peoples’ Research and Engagement Council
